# A Bayesian Model of Perceived Head-Centered Velocity during Smooth Pursuit Eye Movement

**DOI:** 10.1016/j.cub.2010.02.059

**Published:** 2010-04-27

**Authors:** Tom C.A. Freeman, Rebecca A. Champion, Paul A. Warren

**Affiliations:** 1 School of Psychology, Cardiff University, Tower Building, Park Place, Cardiff CF10 3AT, UK; 2 School of Psychological Sciences, University of Manchester, Zochonis Building, Brunswick Street, Manchester M13 9PL, UK

**Keywords:** SYSNEURO

## Abstract

During smooth pursuit eye movement, observers often misperceive velocity. Pursued stimuli appear slower (Aubert-Fleishl phenomenon [1, 2]), stationary objects appear to move (Filehne illusion [3]), the perceived direction of moving objects is distorted (trajectory misperception [4]), and self-motion veers away from its true path (e.g., the slalom illusion [5]). Each illusion demonstrates that eye speed is underestimated with respect to image speed, a finding that has been taken as evidence of early sensory signals that differ in accuracy [4, 6–11]. Here we present an alternative Bayesian account, based on the idea that perceptual estimates are increasingly influenced by prior expectations as signals become more uncertain [12–15]. We show that the speeds of pursued stimuli are more difficult to discriminate than fixated stimuli. Observers are therefore less certain about motion signals encoding the speed of pursued stimuli, a finding we use to quantify the Aubert-Fleischl phenomenon based on the assumption that the prior for motion is centered on zero [16–20]. In doing so, we reveal an important property currently overlooked by Bayesian models of motion perception. Two Bayes estimates are needed at a relatively early stage in processing, one for pursued targets and one for image motion.

## Highlights

► Pursuit-based velocity illusions can be explained by Bayesian inference ► Speed discrimination is worse when stimuli are pursued by eye ► Separate (and early) Bayes estimates are needed for pursued targets and image motion

## Results

**Figure 1:**
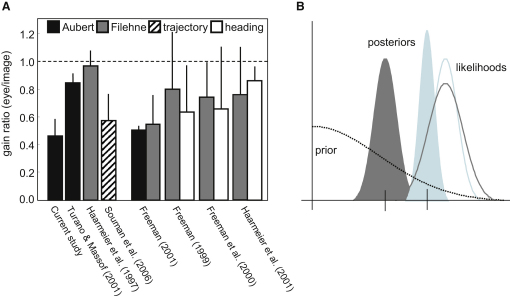
Pursuit-Based Velocity Illusions and Bayesian Inference (A) Summary of recent studies of the Aubert-Fleischl phenomenon, Filehne illusion, trajectory misperception, and perceived heading. The psychophysical data have been converted into a “gain ratio,” which expresses the magnitude of the signals encoding eye velocity with respect to image motion [4, 6, 8, 41]. The four studies on the right directly compared pairs of illusions. Data were taken from figure 3 of [8]; figure 2 of [42]; pp. 69–70 of [4]; figures 4 and 5 of [6]; figure 3 of [41]; figure 7 of [5]; and figure 4 (and T. Haarmeier, personal communication) of [43]. Error bars correspond to 95% confidence intervals. (B) Bayes law applied to motion perception. Perceived speed is determined by the location of the peak of the posterior probability distribution (the maximum a posteriori estimate; short vertical lines). The posterior is the product of a likelihood function (representing sensory evidence) and a prior (representing expectations). A plausible prior for motion perception is that objects are at rest, hence the prior is centered on 0. Noisier sensory signals are less precise and so yield wider likelihood functions (dark curve compared to light curve). The posterior in this case therefore shifts closer to the prior (solid dark curve compared to solid light curve).

Figure 1A demonstrates a consistent finding across a number of studies that eye velocity is often underestimated with respect to image velocity. This finding could reflect differences in the accuracy of underlying motion signals, especially given that sensory information encoding eye speed is likely to be based on motor commands [21] in the situations described in the figure. However, differences in accuracy imply a visual system that has failed to remove systematic errors between early sensory signals, despite evidence that adaptation and calibration lie at the heart of most visual function [22]. The alternative view is that sensory evidence is unbiased (i.e., accurate) but can vary in uncertainty (i.e., precision). In a Bayesian framework, the uncertain sensory evidence is combined with prior expectations about particular properties of the world [12–14]. For motion perception, a plausible prior is that objects are largely at rest [16–20]. The prior is therefore centered on 0, in which case perceived speed decreases as uncertainty rises (Figure 1B). To investigate whether this idea could account for the illusions described in Figure 1A, we measured thresholds for discriminating the speed of pursued and fixated stimuli. At the same time we also measured the size of the Aubert-Fleischl phenomenon in order to see whether the discrimination data could predict the underestimation of eye speed.

**Figure 2:**
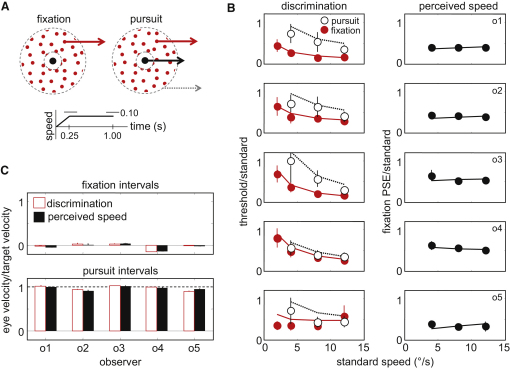
Methods and Main Results (A) Random dot stimuli (0.64 dots/degree^2^, dot diameter = 0.1°, red dots on black background) were viewed in a completely darkened room on a standard cathode ray tube (100 Hz) from a distance of 70 cm. Fixation intervals contained a static fixation target (diameter = 0.2°) and static window (outer diameter = 10°, inner diameter = 2°). Pursuit intervals contained the stimulus, target, and window all moving together, with the start position randomly perturbed by 2°. Motion was ramped over the first 0.25 s and continued at a constant speed for a total duration of 1.0 s. Ramped and constant motions were randomly perturbed by 0.1 s. Discrimination trials and perceived-speed trials were randomly interleaved in the same session. Psychometric functions were obtained via a method of constant stimuli. Cumulative Gaussians were fit to data via maximum likelihood estimation: discrimination thresholds were defined as the standard deviation of the Gaussian, and perceived speed was defined as its mean (point of subjective equality [PSE] for matching fixated tests to pursued standards). (B) Results for five observers. Thresholds and perceived speeds are reported as a fraction of the standard speed. Pursued stimuli were less easy to discriminate than fixated stimuli (left column, open symbols versus closed symbols). Perceived speed slowed during pursuit (right column). Lines show thresholds and PSEs determined by a Bayesian model fit to the raw psychometric data (i.e., they were not fit to the thresholds and PSEs shown in the figure; see [17] for similar strategy). Error bars are 95% confidence intervals, obtained via a bootstrapping technique. (C) Eye movements for fixation intervals (top) and pursuit intervals (bottom). Open bars correspond to discrimination trials, and closed bars correspond to perceived-speed trials. Eye movements were measured with an Eyelink 1000 eye tracker, sampling at 1000 Hz. Trials containing saccades (∼6%) were discarded from eye movement analysis.

In the main experiment, observers judged which of two stimulus intervals (Figure 2A) appeared faster over a series of trials. Fixation intervals (F) consisted of a moving stimulus and a stationary fixation target. The stimulus moved behind a static circular window to ensure that approximately the same region of retina was stimulated during fixation and pursuit. Eye pursuit intervals (E) consisted of the target, window, and stimulus all moving together. The two types of interval were combined in three different ways. Discrimination trials contained F-F or E-E intervals. These two types of trial therefore allowed us to evaluate the precision of signals accompanying fixated and pursued stimuli, respectively. Perceived-speed trials contained E-F or F-E intervals. Trials of this third type allowed us to quantify the size of the Aubert-Fleischl phenomenon because they determined the relative difference in perceived speed between fixated and pursued stimuli.

Figure 2B shows the results of the experiment (symbols) and the model fitting (lines). Each row corresponds to one of the five observers who took part. The discrimination data in the left column show that thresholds were higher for pursued stimuli, meaning that the speed of pursued stimuli was harder to discriminate than the speed of fixated stimuli. Observers were therefore less certain about the sensory evidence defining pursued stimuli, and so their judgments were less precise. The poorer discrimination could have potentially been due to the absence of relative motion, because unlike the fixation intervals, the dot pattern, target, and window all moved together in the pursuit intervals. However, relative motion only influences thresholds at slow speeds [23, 24], a finding we confirmed in a control experiment reported in Figure S1 available online. We therefore conclude that the presence or absence of eye movement is paramount in driving the differences in precision.

According to a Bayesian explanation, greater thresholds during pursuit mean that pursued stimuli should appear slower than fixated stimuli because of the greater influence of the zero-motion prior (Figure 1B). Analysis of E-F and F-E trials supported this prediction (Figure 2B, right column). For all observers, fixated motion needed to be slowed by around 50% to achieve the perceived-speed match. Moreover, the accuracy of eye movements during fixation or pursuit could not explain the data (Figure 2C). The results therefore provide excellent qualitative agreement with the requirements of a Bayesian explanation.

### Model

**Figure 3:**
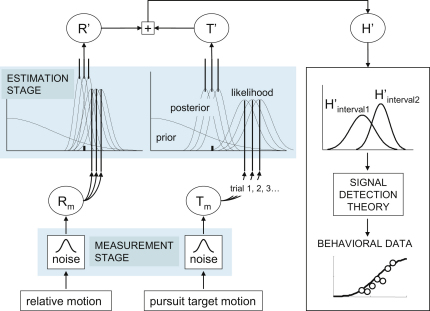
Schematic of the Bayesian Model Measurement stage: separate sources of internal noise are added to unbiased measurements (sensory signals) of relative motion (R_m_) or pursuit-target motion (T_m_). The noise varies as a function of speed and sets the spread of likelihoods. Estimation stage: R′ and T′ are the estimated speeds obtained by multiplying the prior with the likelihood. Their sum yields an estimate of head-centered motion (right). Signal detection theory is used to map the output of the model (e.g., for a two-interval task) onto behavioral performance (i.e., psychometric function).

To determine the extent to which the discrimination data predicts the perceived-speed data, we implemented the Bayesian model shown in Figure 3 (see Supplemental Experimental Procedures for details). The model is designed to account for the type of perceptual errors represented by the four illusions identified in Figure 1A. It therefore recovers perceived motion with respect to the head (as opposed to motion with respect to the scene; see [25]). In keeping with the consistency between illusions, the model does not differentiate between how image motion and eye motion are arranged in time. It therefore does not care whether the two motion types are compared consecutively (the Aubert-Fleischl phenomenon) or simultaneously (the Filehne illusion, trajectory misperception, slalom illusion).

The model differs from traditional accounts of head-centered motion perception in two important ways. First, it treats motion measurements and motion estimates separately. In comparison, traditional accounts assume that motion estimates are based on sensory signals alone. Second, it emphasizes the role of relative image motion (between background object and target) and pursuit target motion. Traditional accounts emphasize absolute retinal motion and eye velocity. The eye movements were extremely accurate in our experiments, so relative motion was approximately equal to absolute image motion, and target motion was equal to eye velocity. But not all observers do so well—for instance, pursuit slows by around 10%–20% between 20 and 60+ years of age [26]. The model is therefore designed to account for situations in which pursuit is inaccurate and imprecise (see Discussion).

The model consists of a measurement stage (Figure 3, bottom left) and an estimation stage (Figure 3, top left). The measurement stage contains separate internal noise sources, one for relative motion (R) and one for pursuit target motion (T). The standard deviation of the internal noise was defined as σ(v) = av^b^ + c (see [27, 28] and Supplemental Experimental Procedures). The estimation stage implements separate Bayes estimators for R and T via the same principle described earlier in Figure 1B. The model assumes two priors, both centered on a speed of 0. The priors represent the observer's expectations about target motion and relative motion. They are both based on the assumption that objects tend to be at rest. In the absence of reliable sensory information, the observer expects target objects to not move and hence for there to be no relative motion between one object (the target) and another.

The inputs to the estimation stage (the sensory measurements R_m_ and T_m_) vary over intervals and trials as a result of internal noise. Hence, the Bayes estimates R′ and T′ vary as well, as does their sum H′, which yields the observer's estimate of head-centered motion. For a two-interval task, two distributions of H′ are produced, one for each interval (Figure 3, right). Straightforward application of signal detection theory links the model to data. It does so by combining the two intervals into a single “decision” variable. The probability of choosing one interval over the other can then be determined. For discrimination, the two intervals consist of image motion or eye motion (plus the small contribution of the noise term “c” when the input is 0). For the perceived-speed condition, the decision variable combines the two different types of motion. The combination is therefore independent of temporal order, reiterating the fact that how the different types of motion are arranged in time does not matter to the model.

Figure 2B shows that thresholds were approximately constant for faster speeds when expressed as a proportion of the standard. This is known as Weber's law and is thought to reflect an early nonlinearity in the coding of speed, combined with fixed internal noise (i.e., noise independent of speed) [17, 29, 30]. However, as analogous work on contrast discrimination has shown [31, 32], similar results can be obtained if an early nonlinearity is combined with variable noise, a point we confirmed in earlier implementations of our model. Our data also show that Weber's law breaks down at slow speeds, a finding well known for image-motion processing [33] and one that our results now extend to pursued stimuli. To account for this latter behavior, the early nonlinearity can be augmented in a number of ways (see [17] for an example). However, from a Bayesian perspective, it turns out that early nonlinearities may not be necessary. Using a combination of variable internal noise and a zero-motion prior, we were able to reproduce the thresholds we found very well, as can be seen by comparing the model and data in Figure 2B. Indeed, invoking early nonlinearities presents problems for Bayesian accounts of head-centered motion perception. If different nonlinearities for R and T were used, the Bayes estimation stage is unnecessary—changes in velocity estimates would be captured by differences in signal accuracy that result from the separate nonlinearities at the measurement stage (see [6, 8] for demonstration). The alternative is to enforce identical nonlinearities. But this is unlikely, given that the measurements of image motion and eye velocity are based on different types of motion signal.

**Figure 4:**
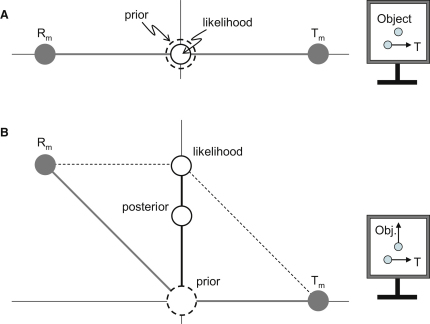
Unbiased Sensory Measurements Cannot Lead to Pursuit-Based Velocity Illusions (A) No Filehne illusion could result if sensory measurements were biased because pursuit over a stationary background produces relative motion that is equal and opposite to the eye movement. The unbiased sensory signals R_m_ and T_m_ must therefore be equal and opposite, too. Their sum defines the location of the putative head-centered likelihood and is centered on 0. Given that the prior is centered on 0, too, the posterior (not shown) must be situated there as well. Stationary objects would always appear stationary. (B) Using similar reasoning, no misperception of trajectory could result because the putative head-centered likelihood is located in the true direction. In this example, pursuit to a target T is made over a vertically moving object. This produces relative motion that is oblique. The sum of unbiased measurements of R and T defines the location of putative head-centered likelihood and lies in the correct direction (upward, black vertical line). If the prior is centered at 0, the posterior can only ever be located in the correct direction (vertical for this example), at a speed dependent on the spread of the likelihood.

The second way in which the model differs from previous Bayesian accounts of motion perception is the use of two estimators based on separate likelihoods for R and T. An alternative is to sum signals at the measurement stage and so yield a single head-centered likelihood. However, this could never produce a Filehne illusion, assuming that the signals are unbiased. When smooth pursuit is made over a stationary background, observers typically report that the background appears to move against the eye movement. In this situation, pursuit produces equal and opposite motion in the image (Figure 4A). Hence, the sum of unbiased measurements of eye motion (T_m_) and image motion (R_m_) must be centered on 0. Importantly, the sum defines the location of the putative head-centered likelihood. Given that the prior is also centered on 0, the posterior distribution defining the observer's estimate must be, too, so no Filehne illusion can result. Similar reasoning shows why trajectory misperception cannot occur (Figure 4B). Of course, one way to fix this alternative Bayesian account is to introduce biases into the initial sensory measurements. But as discussed above, this eliminates the need for a Bayes estimation stage. We conclude that in order to account for pursuit-based velocity illusions, two Bayes estimates are needed, one for R and one for T.

For modeling purposes, we assumed that the two priors had the same standard deviation, a justifiable assumption given that both depend on the idea that objects tend to be at rest. The estimation stage therefore consisted of a single parameter (the standard deviation of the prior), whereas the measurement stage consisted of six (one set each of three internal noise parameters for R and T). The seven-parameter model was fit simultaneously to all ten psychometric functions of each observer via a maximum-likelihood technique. The lines in Figure 2B show the model's discrimination thresholds and perceived speeds. The model predicts the data extremely well. Allowing the priors to have different standard deviations would not substantially improve the fit.

## Discussion

The new Bayesian model of head-centered motion perception presented here is able to explain a range of pursuit-based velocity illusions. The model is based on the idea that sensory signals encoding the speed of eye motion and image motion differ in precision, not accuracy. The model raises a number of issues that need to be considered when applying Bayes theory to motion perception. First, the model emphasizes the role of pursuit-target motion and relative motion, in part because this formulation guarantees that the priors are properties of the world. Second, the Bayes estimates must be made before information about eye motion and image motion is combined, otherwise no illusions can result. Third, separate Bayes estimates for relative motion and pursuit-target motion are required to explain these illusions. Finally, the combination of unbiased signals, variable internal noise, and a prior centered on zero is sufficient to predict the discrimination performance we found.

The Aubert-Fleischl phenomenon is known to decline as the contrast of fixated stimuli is lowered [34]. Because contrast does not affect the perceived speed of pursued stimuli, the change to the Aubert-Fleischl phenomenon must be driven solely by the lower estimates of image motion brought about by decreasing contrast. This effect is easy to capture in the model because manipulating contrast is one way of influencing the precision of image motion measurements—indeed, the effect of contrast provides the bulk of the evidence supporting Bayesian models of motion perception [17, 19, 20] (although see [35] for evidence that the relationship between perceived speed and contrast may be more complex than often described; see also [36]). At present it is unknown whether contrast influences the Filehne illusion, trajectory misperception, or slalom illusion in a similar way. It may be that variations in stimulus dimensions like contrast help explain why the degree of underestimation of eye speed varies across the studies shown in Figure 1A.

Unlike traditional accounts of head-centered motion perception, our new model emphasizes the role of relative motion and pursuit-target motion. There are good empirical reasons for doing so. Recent evidence suggests that relative motion overrides the use of absolute retinal motion during pursuit, even when trial-by-trial feedback is given on the latter [37]. Relative motion therefore appears paramount for these types of motion judgment. The use of pursuit-target motion may stem from the fact that pursuit eye movements are not always accurate and also vary over time [26, 38]. Hence, estimating target motion solely on the basis of extraretinal eye velocity information [21] would be subject to the same inaccuracies and temporal variability. These are easily offset by adding localized image motion information related to the movement of the pursuit in the image (retinal slip), an idea supported by the recent finding that discriminating the motion of pursued targets is best predicted by the combination of retinal slip and eye velocity [39] (see also evidence of cells in the medial superior temporal area that respond to eye velocity and retinal slip [40]). Indeed, the combination of slip and eye velocity may explain why the sensory measurement of pursuit-target motion is less precise than corresponding measures of relative motion. By summing eye velocity information with retinal slip, the internal noise related to pursuit-target motion originates from two disparate sources. The internal noise related to relative motion, however, derives from a single source, namely the retinal image. On many occasions, this single noise source can be reduced further by integrating over larger areas.

## Supplemental Information

Document S1. Supplemental Experimental Procedures, Three Figures, and One Table
