# Supplementary material for: A Bayesian Model of Perceived Head-Centered Velocity during Smooth Pursuit Eye Movement
Source: Curr Biol. 2010 Apr 27;20(8-6):757–62. doi: 10.1016/j.cub.2010.02.059 (PMC2861164; doi:10.1016/j.cub.2010.02.059)
Supplement: Document S1. Supplemental Experimental Procedures, Three Figures, and One Table [file mmc1.pdf]

Current Biology, Volume *20*

Supplemental Information

A Bayesian Model of Perceived Head-Centered Velocity during Smooth Pursuit Eye Movement

Tom C.A. Freeman, Rebecca A. Champion, and Paul A. Warren

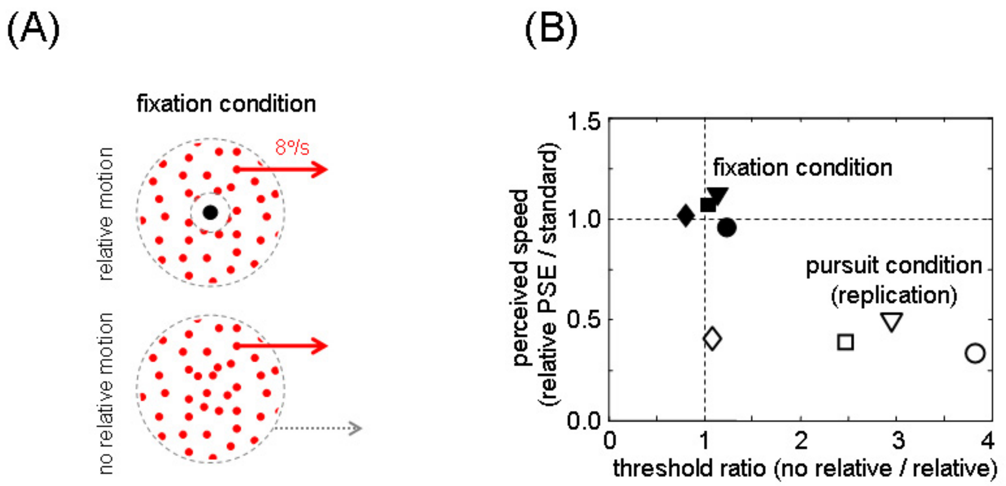

Figure S1.
Results of a control experiment showing that the presence or absence of relative motion is not the reason why pursued stimuli in the main experiment appeared slower than fixated stimuli. Different symbol shapes correspond to different observers. See text for details.

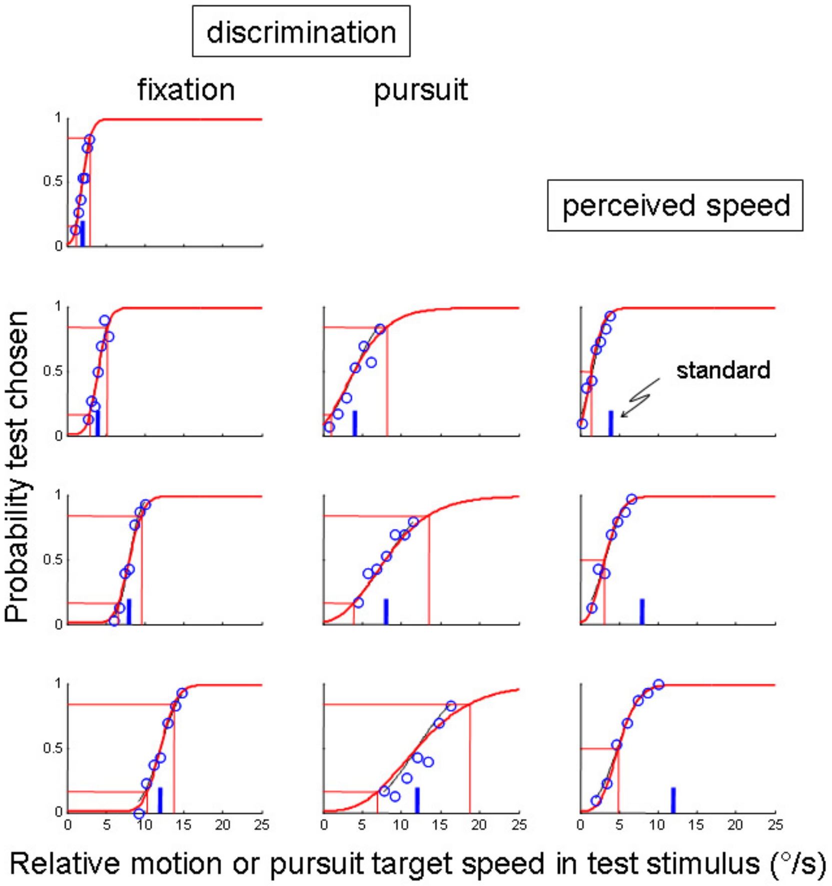

Figure S2.
Representative observer's raw psychometric data with associated model fit (red lines). The model has 7 parameters and these were fit simultaneously across all 10 graphs (see text for details). First two columns correspond to discrimination trials and third column perceived-speed trials. Black curves show standard psychometric functions fit to individual panels. In most cases these are hidden by the model curves.

Table S1. Best-Fitting Parameter Values for Each of 5 Observers

	Internal noise						Prior
Observer	Relative motion (R)			Pursuit target motion (T)			
	a	*b*	*c*	*a*	*b*	*c*	σ
o1	0.14635	0.31107	0.13808	0.04384	0.57662	0.40757	0.01186
o2	0.10223	0.34886	0.05690	0.13364	0.30253	0.08618	0.00432
o3	0.36452	0.08794	0.20993	0.31443	0.06663	0.48642	0.06553
o4	0.10415	0.53404	0.17715	0.06329	0.56682	0.40804	0.02205
o5	0.12062	0.22516	0.06325	0.20534	0.19806	0.00460	0.01451

Supplemental Results

Control Experiment

To investigate the influence of relative motion in the results of our main experiment, we compared discrimination and perceived speed for two types of fixation interval shown in Figure S1A. One contained relative motion as before and one contained no relative motion. The results in Figure S1B (closed symbols) showed that the ability to discriminate the speed in the two fixation intervals was the same: the threshold ratio clusters around 1. In fact, this turns out to be a particularly strong test of Bayesian accounts of motion perception, because the absence of a discrimination difference should lead to equal perceived speeds. Again, the closed symbols show that the perceived speed ratio for these two intervals was also close to 1.

To achieve fixation without relative motion, we reduced interval duration to 0.25s. This prevented any significant pursuit eye movement, while simultaneously allowing the presentation of moving stimuli without any stationary references (such as a stationary fixation point and static window). The comparison fixation intervals, those that contained relative motion, had a similarly short duration. Unfortunately, displaying stimuli this briefly meant that short fixation intervals could not be compared to short pursuit intervals – for the same reason that short durations prevent eye movements during fixation, they also prevent pursuit. The closest comparison possible was to repeat the equivalent long-duration conditions of the experiment in the main paper. In doing so, we replicated our original finding (open symbols).

Supplemental Experimental Procedures

Derivation of Model

In the main paper, two velocities (relative motion R and pursuit target motion T) serve as input to the Bayesian model. Here we formalise the model by considering first how a generic velocity ($\mathbf{v}$) is estimated by the Bayesian observer. We then show how this generic estimate is used to estimate head-centred motion, that is, the sum of separate Bayes estimates of R and T. Finally we show how the collection of estimates maps on to behavioural data (ie. a psychometric function).

Measurement Stage

Velocity v is measured by an unbiased sensory signal with a mean of v. The signal is corrupted by Gaussian internal noise with a mean of 0 and standard deviation given by:

$$\sigma_{\text{int}}(\mathbf{v}) = a\mathbf{v}^{b} + c \qquad (1)$$

The nonlinear portion of this equation is similar to that used previously to describe the response variance of cortical cells. Britten et al (1993) investigated the response of cells in motion area MT and found their variance proportional to the mean (i.e. Poisson noise). Hence standard deviation varies as the square-root (i.e. b=0.5 – see also Dakin et al, 2005). In our model fits we found values for b in reasonable agreement (mean = 0.32: see Table S1). Equation (1) also defines a parameter ‘c’ that represents the internal noise when v=0.

Estimation Stage

Internal noise makes the sensory signal vary from trial to trial. Bayes law allows the observer to draw sensible inferences about the probable state of the world (e.g. the degree of relative motion or pursuit target motion). Uncertain sensory evidence is combined with prior expectations to yield a posterior probability equal to a likelihood function multiplied by the prior (divided by a normalisation factor). The likelihood defines (in part) the variability of the sensory evidence. The location of the peak of the posterior is typically taken as the perceptual estimate (the MAP estimate). Using the symbol '~' to denote 'is distributed as', then assuming that the prior $P \sim N(\mu_P, \sigma_P^2)$ and the likelihood $L \sim N(\mu_L(v), \sigma_L^2)$, it can be shown that the posterior S is also normally distributed with mean $\mu_S(\mathbf{v})$ and variance $\sigma_S^2(\mathbf{v})$ (see Ma et al, 2006):

$$\mu_S(\mathbf{v}) = \frac{\mu_L(\mathbf{v})\sigma_P^2 + \mu_P\sigma_L^2(\mathbf{v})}{\sigma_P^2 + \sigma_L^2(\mathbf{v})} \qquad (2)$$

$$\sigma^2{}_S(\mathbf{v}) = \frac{\sigma_P^2\sigma_L^2(\mathbf{v})}{\sigma_P^2 + \sigma_L^2(\mathbf{v})} \qquad (3)$$

Note that for an unbiased estimator, $\mu_L(v) = v$. We assume that stochastic internal noise is the only reason that perceived speed varies across intervals and trials. Each time the observer estimates velocity $\mathbf{v}$, the mean of the likelihood in Equation (2) is therefore shifted by the internal noise of the measurement (Stocker & Simoncelli, 2006). Consequently we assume that the mean of the likelihood is actually a random variable $\mu'_L(\mathbf{v})$ corrupted by Gaussian noise $n \sim N(0, \sigma_{\text{int}}^2(\mathbf{v}))$. In other words:

$$\mu'_L(\mathbf{v}) \sim N(\mu_L(\mathbf{v}), \sigma_{\text{int}}^2(\mathbf{v})) \qquad (4)$$

Note that in general, the standard deviation of the likelihood $\sigma_L(\mathbf{v})$ incorporates both internal noise and external uncertainty. The latter term corresponds to factors such as ambiguities over the projection of three dimensions to two. In the model fits we assumed there was no external uncertainty (ie. $\sigma_{ext} = 0$) but, for completeness, we derive the model with external uncertainty included:

$$\sigma_L^2(\mathbf{v}) = \sigma_{\text{int}}^2(\mathbf{v}) + \sigma_{ext}^2 \qquad (5)$$

Speed discrimination is determined by the way the mean of the posterior μ_S changes across intervals and trials. More formally, due to its dependence on $\mu'_L(\mathbf{v})$, the mean of the posterior is the random variable μ'_S defined by:

$$\mu_S'(\mathbf{v}) = \frac{\mu_L'(\mathbf{v})\sigma_P^2 + \mu_P\sigma_L^2(\mathbf{v})}{\sigma_P^2 + \sigma_L^2(\mathbf{v})} \qquad (6)$$

Consequently, the individual MAP estimates form their own probability distribution. Crucially, we can derive the mean and variance of this probability distribution as follows. We first note that Stocker & Simoncelli (2006) reverse engineered the motion prior from psychophysical data and provided good support for the assumption that it has a mean of zero. Setting μ_p to zero in Equation (6) we obtain:

$$\mu_S'(\mathbf{v}) = \frac{\mu_L'(\mathbf{v})\sigma_P^2}{\sigma_P^2 + \sigma_L^2(\mathbf{v})} \qquad (7)$$

Next we make use of standard results from mathematical statistics. For a random variable *X* and scalar *r,* the expected value and variance of the random variable *rX* are given by:

$$E[rX] = rE[X]$$
$$Var[rX] = r^2 Var[X]$$

Since Equation (7) tells us that $\mu_S'(\mathbf{v})$ is a linear function of the random variable $\mu_L'(\mathbf{v})$ of which we know the mean and variance (see Equation (4)), we use the above results and substitute Equation (5) to obtain:

$$E[\mu_S'(\mathbf{v})] = \frac{\mu_L(\mathbf{v})\sigma_P^2}{\sigma_P^2 + \sigma_{\text{int}}^2(\mathbf{v}) + \sigma_{ext}^2} \qquad (8)$$

$$Var[\mu_S'(\mathbf{v})] = \left(\frac{\sigma_P^2}{\sigma_P^2 + \sigma_{\text{int}}^2(\mathbf{v}) + \sigma_{ext}^2}\right)^2 \times \sigma_{\text{int}}^2(\mathbf{v}) \qquad (9)$$

Estimating Head-Centred Motion

Head-centred motion is the sum of eye velocity and retinal motion or, equivalently, pursuit target motion (T) and relative motion (R). Arguments in favour of the latter are presented in the main paper, but note this only becomes an important issue when eye movements and/or fixation are inaccurate. The Bayesian observer modelled here therefore estimates head-centred motion by adding separate estimates of T and R with means and standard deviations defined by Equations (8) and (9). Recall that for an unbiased estimator, $\mu_L(v) = v$. In this case, the posterior for head-centred motion (H) has a mean and variance:

$$E[\mu_H'(\mathrm{T},\mathrm{R})] = \frac{\mathrm{T}\sigma_P^2}{\sigma_P^2 + \sigma_{\text{int}}^2(\mathrm{T}) + \sigma_{ext}^2} + \frac{\mathrm{R}\sigma_P^2}{\sigma_P^2 + \sigma_{\text{int}}^2(\mathrm{R}) + \sigma_{ext}^2} \qquad (10)$$

$$Var[\mu'_H(\mathrm{T},\mathrm{R})] = \left(\frac{\sigma_P^2}{\sigma_P^2 + \sigma_{\mathrm{int}}^2(\mathrm{T}) + \sigma_{ext}^2} \right)^2 \times \sigma_{\mathrm{int}}^2(\mathrm{T}) + \left(\frac{\sigma_P^2}{\sigma_P^2 + \sigma_{\mathrm{int}}^2(\mathrm{R}) + \sigma_{ext}^2} \right)^2 \times \sigma_{\mathrm{int}}^2(\mathrm{R}) \quad (11)$$

where $\sigma_{\mathrm{int}}^2(\mathrm{T})$ and $\sigma_{\mathrm{int}}^2(\mathrm{R})$and are the internal noises associated with T and R, respectively. They are defined by two different sets of values for a, b, c (see Equation (1)).

Linking Model to Psychometric Function

Using standard signal detection theory, performance in a two interval task is governed by:

$$d' = \sqrt{2}\,\frac{E[\mu'_H(\mathrm{T}_1,\mathrm{R}_1)] - E[\mu'_H(\mathrm{T}_2,\mathrm{R}_2)]}{\left[Var[\mu'_H(\mathrm{T}_1,\mathrm{R}_1)] + Var[\mu'_H(\mathrm{T}_2,\mathrm{R}_2)]\right]^{1/2}} \quad (12)$$

where $(\mathrm{T}_1,\mathrm{R}_1)$and $(\mathrm{T}_2,\mathrm{R}_2)$ are the velocities in the two intervals. Note that in our experiments, $\mathrm{R}_\mathrm{i} = 0$ in pursuit intervals and $\mathrm{T}_\mathrm{i} = 0$ in fixation intervals. Hence the expectation $E[\mu'_H(\mathrm{T},\mathrm{R})]$ includes one likelihood only. However, this is not the case for the associated variances because Equation (1) defines a constant 'c' corresponding to the noise at $\mathrm{R}_\mathrm{i} = 0$ or $\mathrm{T}_\mathrm{i} = 0$. The variance of each interval therefore combines internal noise from both R and T estimates.

For a range of velocities $\mathbf{v}_1$(the 'test') about $\mathbf{v}_2$ (the 'standard'), the probability of choosing one interval over the other is determined by:

$$P = \Phi\left(d'/\sqrt{2}\right) \quad (13)$$

where Φ denotes the cumulative distribution function of the standard normal distribution. In the model fitting we included a lapse rate parameter g as suggested by Wichman & Hill (2001). The lapse rate captures those instances when the observer overlooks one or other interval and is forced to guess:

$$P_g = (1-g)P + 0.5g \quad (14)$$

The parameter *g* was fixed for all observers at 0.02.

Model Fitting

The model was implemented in MatLab (Mathworks Inc.) using their simplex search utility fminsearch. Figure S2 shows the raw data (symbols) for one observer, together with model's psychometric functions (thick red lines) as well as the fitted psychometric functions (thin black lines) that summarise observer thresholds and perceived speeds in main paper (symbols in Figure 3B of main text). The thin black lines are mostly hidden by the fitted model. There are four

standard speeds for fixation discrimination (left column), three standards for pursuit discrimination (middle) and three standards for perceived-speed (right). Best-fitting values for the model's parameters are given in Table S1. The model consists of seven parameters: three internal noise parameters for R and three for T as defined by Equation (1), together with one standard deviation for the prior. Maximum likelihood estimation was used to fit the seven parameters simultaneously across all 10 graphs. This was done separately for each observer. Model thresholds and PSEs were obtained using spline interpolation. Because the psychometric functions for the model are not symmetric about the PSE, thresholds were defined as 0.5[v(0.84)-v(0.16)], where v(P) is the test speed interpolated at the probability P. The asymmetry arises in part because of the use variable internal noise defined in Equation (1).
